# Malignant transformation of endometriosis in a laparoscopic trocar site a case report

**DOI:** 10.1186/s12905-022-01749-3

**Published:** 2022-05-13

**Authors:** Ling Han, Bingyi Zhang

**Affiliations:** 1 Department of Obstetrics and Gynecology, The People’s Hospital of China Three Geoges University The People’s Hospital of China Three Gorges University. The First People’s Hospital of Yichang, Jiefang Road 4, Yichang City, 443003 Hubei Province People’s Republic of China; 2 Department of Ultrasound Imaging, The People’s Hospital of China Three Gorges University. The First People’s Hospital of Yichang, Yichang City, Hubei Province People’s Republic of China

## Abstract

**Background:**

Malignant transformation of endometriosis is infrequent at the laparoscopic trocar site. Although malignant transformation is uncommon, it must be acknowledged in order to achieve radical resection.

**Case presentation:**

We report on a 54-year-old woman with trocar site endometriosis 2 years after laparoscopic ovarian endometrial resection. Physical examination revealed a subcutaneous solid tumor with a diameter of 3 cm surrounding the scar of laparoscopic surgery in the right lower abdomen. Transabdominal ultrasonography showed a cystic tumor in the subcutaneous adipose layer of the right lower abdomen. The pathological diagnosis was poorly differentiated endometrioid carcinoma. Hysterectomy, bilateral salpingo-oophorectomy and pelvic lymphadenectomy were then performed. Histological examination revealed mixed endometrioid carcinoma and clear cell carcinoma. After six cycles of chemotherapy, computed tomography showed no signs of recurrence.

**Conclusions:**

Malignant transformation of laparoscopic endometriosis is very uncommon, and the diagnosis and stage are determined by clinical manifestations and imaging examination. The main therapy methods are radical surgery combined with neoadjuvant chemotherapy and adjuvant radiotherapy. At the same time, reducing iatrogenic abdominal incision implantation is an effective prevention method.

## Background

The prevalence of endometriosis in the general population is about 10% of women of reproductive age. Endometriosis refers to abnormal growth of endometrial tissue that survives outside the uterus and occurs after cesarean section, laparoscopic surgery, hernia repair and other operations. The most common sites are rectovaginal septum, colon and vagina. The implantation of ectopic endometrium into scar tissue of the abdominal wall is called abdominal endometriosis [1]. At present, with the increase in the rate of cesarean section, the incidence of scar endometriosis has also risen. Scar endometriosis is a potential complication of cesarean section, with an incidence of about 0.03–0.45% [2, 3]. However, malignant transformation of abdominal endometriosis is very uncommon, the incidence is less than 1%, and the early diagnosis is very difficult [4]. We report a case of malignant transformation of trocar site endometriosis after laparoscopic ovarian endometrial resection, providing a scientific basis for the diagnosis and treatment of abdominal endometriosis.

## Case presentation

We report a case of a 54-year-old woman who presented with a new mass on the abdominal wall scar that had grown in the past 3 months. She had a cesarean sections 31 years ago. In 2017, she underwent laparoscopic right adnexectomy for a chocolate cyst of the right ovary.

**Fig. 1:**
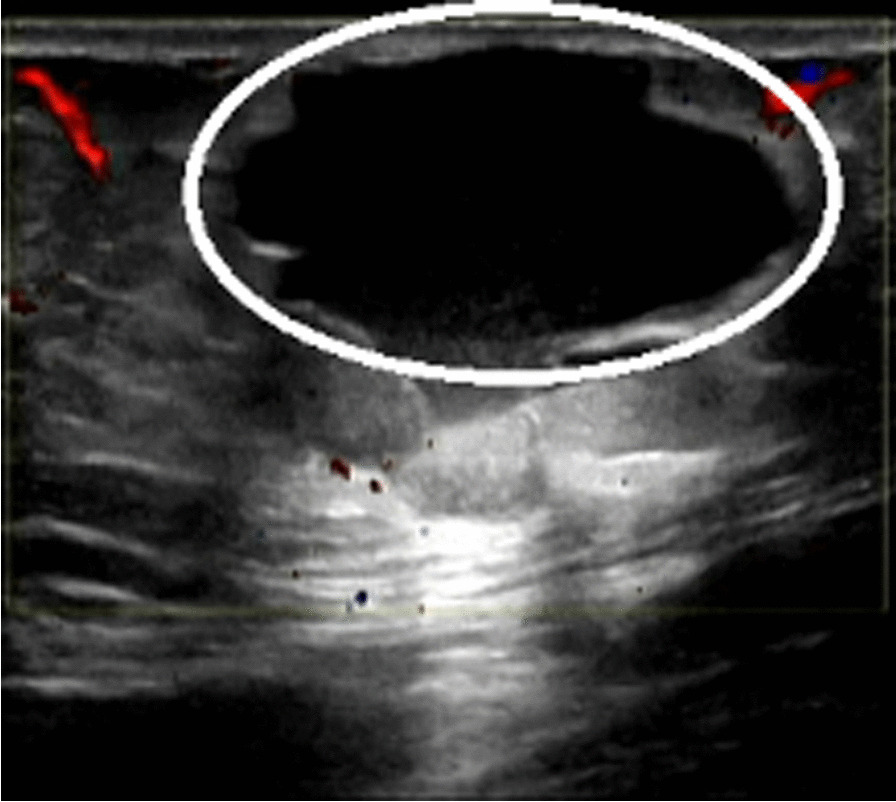
Ultrasound image showed cystic mass in subcutaneous fat layer of right lower abdomen (3.1 × 2.3 cm)

**Fig. 2:**
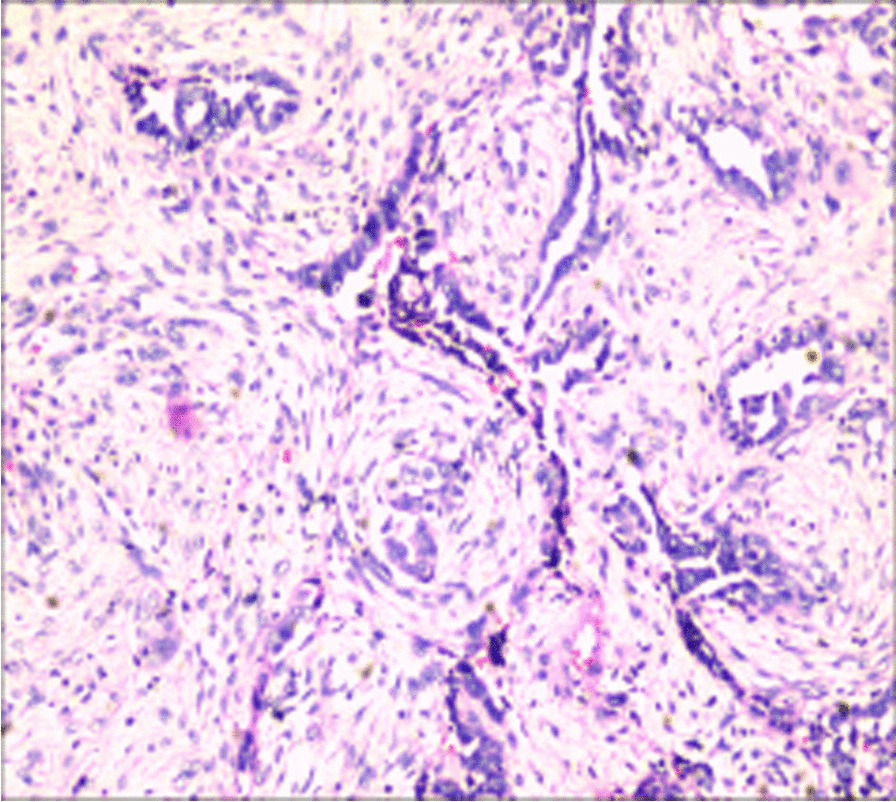
Poor differentiated endometrioid carcinoma arising in a foci of end endometriosis in the laparoscopic trocar site scar

An increased abdominal mass was reported in our hospital, and physical examination revealed a subcutaneous solid tumor with a diameter of 3 cm around the scar of laparoscopic surgery on the right lower abdomen. Transabdominal ultrasound showed a cystic tumor in the subcutaneous adipose layer of the right lower abdomen (Fig. 1). Trans-vaginal ultrasonography showed normal findings.Routine laboratory findings and tumor markers (AFP, CA125, HE4 and CEA) were normal, but serums CA-199 were elevated at 41.48U/m U/mL. The patient was excised under general anesthesia. During the intraoperative exploration of the fat layer, a 3 × 2 cm mass with hard texture could be reached. After the fat was cut open, a cystic mass could be seen, in which coffee-colored fluid flowed out a poorly differentiated endometrioid carcinoma (Fig. 2). PAX-8, ER, KI-67 and CD10 were positive, while STATB2, GATA3, CDX-2 and PR were negative. Combined with the patient's history of laparoscopic surgery, ultrasound and pathological findings, it was highly suspected that the tumor was a malignant transformation of laparoscopic trocar endometriosis.

Whole-body positron emission tomography and computed tomography (18-FDG PET-CT) were performed at disease stage 14 days after surgery and showed slight hypermetabolism in the incision area. Multiple swollen lymph nodes in retrodiaphragmatic, retroperitoneal, bilateral iliac vessels and right inguinal area, abnormal metabolism.

Our patient then underwent surgical staging, laparoscopy through the peritoneum. The abdominal and renal pelvis were carefully examined, intraoperative adhesions between the greater omentum, the abdominal wall and the fundus of the uterus were observed. Tumor nodules were observed on the surface of the sigmoid colon and rectum. Right total iliac, external iliac, internal iliac, obturator lymph nodes and para-aortic lymph nodes were enlarged and fused into masses. Final pathology revealed a metastatic poorly differentiated adenocarcinoma arising from a foci of endometriosis.Tumor metastasis was observed in right pelvic lymph node and right inguinal lymph node.The remainder of the specimens were negative for malignancy.

Serum CA-125 and CA-199 increased to 36 one month after operation, 48 U/mL and 77.67U/mL, respectively. Serum CA-125 and CA-199 decreased to normal 4 months after surgery. After the operation, patients received systemic chemotherapy (6 cycles of tri-weekly liposomal doxorubicin 30 mg/m2 and carboplatin AUC = 5), which is similar to adjuvant chemotherapy for epithelial ovarian cancer. CT scans showed no evidence of recurrence after 6 cycles of chemotherapy. Three months after 6 times of chemotherapy, she underwent repeat 18-FDG PET-CT examination, which revealed most of the original enlarged lymph nodes disappeared, with only a few small lymph nodes behind the peritoneum and low metabolism.Our patients showed no signs of recurrence 16 months after initial diagnosis,CA199, AFP, CA125, HE4 and CEA were all normal.

## Discussion and conclusions

Abdominal wall endometriosis (AVE) is a relatively rare condition that normally occurs on surgical scars. All patients who have undergone pelvic surgery are at risk for this disease, which is mostly associated with cesarean section, laparoscopic surgery, followed by uterine surgery. A small number of patients with no history of abdominal surgery may also have this disease. The spread of endometrial tissue or endometriosis at the time of surgery is biologically credible because there is an opportunity to inoculate endometrial cells from hysterotomy or endometrioma into the peritoneum or abdominal wall. Malignant transformation of abdominal wall endometriosis and endometriosis at the trocar site after laparoscopic surgery are extremely rare and may result from pneumoperitoneum or may result from direct contact of the excised endometriosis lesion with the abdominal wall. Malignant transformation of abdominal wall endometriosis is very rare and easily overlooked by clinicians. Diagnosis is usually late, leading to missed opportunities for optimal treatment. These require us to diagnose and treat them timely and appropriately.

### Histopathology

It has been reported that the most common histologic types of endometriosis are clear cell carcinoma and endometrioid carcinoma, including clear cell carcinoma was 63–66.7%, followed by endometrioid carcinoma with 14.6–22% [5, 6]. Another case of mixed tumor of clear cell and endometrioid carcinoma was reported [7]. In 1925, Sampson described the malignant transformation of the first case of endometriosis, and he submitted three diagnostic criteria: (1) there is both tumor tissue and endometriosis in the tumor, (2) the histologic appearance is identical to the characteristic endometrial stroma surrounding glands, (3) there is no other primary tumor site. In addition, Scott added the fourth criterion of metaplasia between benign endometriosis and cancer in 1953, but this suggestion remains to be discussed [8]. CD10 is a reliable and sensitive (88%) immunohistochemical marker of endometrial stroma and is expressed in a small number of cytogenic chorionic cells, greatly supporting the diagnosis [9].

### The etiopathogenesis of endometriosis

Recent studies have confirmed that endometriosis has many etiological variables, with genesis in genetics, epigenetic, immunology, endocrinology, and microbiome. There are some theories about the causes and pathogenesis of endometriosis: Treloar's 1999 study of the Australian population proved a 2:1 concordant ratio between identical and fraternal twins, with a corresponding genetic risk of 2.34 affecting microbial sisters. The results indicate that 51% of endometriosis is caused by genetic factors [10]. The increased risk of inheritance in first-degree relatives (5–8%) indicates polygenic and multifactorial inheritance rather than single-gene inheritance [11]. D’Alterio et al. discovered that uterine contractile force is affected by the imbalance of female genital tract (FGT) microbiota and the corresponding inflammatory response, and can increase the adhesion of relevant endometrial cells in retrograde menstruation and peritoneal cavity [12]. They also discovered higher concentrations of IL-17 in peritoneal fluid in patients with endometriosis than in patients without endometriosis.

IL-17 activates pro-angiogenic cytokines such as IL-8,—1 ß. Consequently, IL-17 plays a critical role in the pathogenesis of endometriosis by promoting ectopic endometrial tissue survival, implantation and proliferation through the proliferation of blood vessels on the peritoneal surface [12]. In addition, dysfunctional natural killer cells (NK) inhibit the phagocytic activity of macrophages, clear the expression of receptors, and induce Treg lymphocytes, whose activity is inhibited so that endometrial cells can escape peritoneal immune surveillance [13, 14]. Lagana et al. found that M1 and M2 macrophages were substantially higher in the endometriosis group than in the control group. From stage I to stage IV of endometriosis, M1 macrophages gradually decrease and M2 macrophages gradually increase, which may lead to proinflammatory microenvironment disease in the early stage of inflammation and profibrotic activity in the late stage [15]. Pagliardini et al. proved that a 21 KB single nucleotide polymorphism (SNP) rs7521902 located upstream/downstream of WNT4 region has a susceptibility site for endometriosis [16]. WNT4 is also expressed at the peritoneal level and promotes the conversion of peritoneal cells to endometrial cells through a pathway that plays a part in the development of the female genital tract [17].

A GWAS investigated 7090 individual samples (2594 patients with endometriosis and 4496 healthy controls) and identified a high-risk marker for endometriosis: SNP RS3820282 located in the WNT436 gene region [18]. Sequences of the regulatory genes ESR1 and ESR2 may be the main candidate genes for the occurrence of endometriosis and ovarian cancer [18].

### Clinical feature

The typical clinical manifestation of malignant transformation of abdominal endometriosis is a combination of mass, pain and circulating signs.These favorable signs are beneficial to the diagnosis, but not all cases are typical symptoms. Ivka et al. reported that these symptoms continued for eight years and were exacerbated in the last six months before surgery. More than 50% of cases have no pain connected to the menstrual cycle [19]. Ecker et al. studied 65 patients whose primary clinical manifestation was abdominal pain, with abdominal mass accounting for 73.8% and abdominal mass for 63.1% over a 12-year period [20]. Ozel et al. reported that 100% of the patients had abdominal mass. 83.3% incidence of pain, recurrent pain was 73.3%, aperiodic pain was 26.6% [21]. Scar enlargement is a common phenomenon during menstruation. Between 10 and 50% of preoperatively diagnosed cases are accurate [22, 23]. Clinically, if the symptoms such as dyspareunia or dysmenorrhea worsen or progress, especially when the realistic mass and serum HE4 level is elevated, attention should be paid to the possible progression of ovarian cancer [24]. In addition, inguinal lymph node is the most common tumor metastasis and recurrence of parts, possibly because the superficial abdominal wall lymphatic vessels located below the umbilicus flow to the superficial inguinal lymph nodes [25].

### Image examination

Preoperative ultrasound, pelvic MRI and other imaging tests are important in diagnosing malignant transformation of abdominal endometriosis. The ultrasonographic appearance of AWE can vary considerably. The most common discovery is a cystic, polycystic, solid or mixed nodules close to the cesarean scar, with irregular boundary, heterogeneous texture characterized by scattered internal hyperechogenic foci, peripheral hyperechogenic ring and scanty vascularity [26]. Further MRI examination is recommended in this case. AWE usually presents as a well-defined solid or mixed mass with contrast enhancement, often accompanied by bleeding. CT examination may also be considered to preliminarily determine the benign and malignant lesions [27]. In highly suspected malignancies, PET-CT should also be used to determine disease stages.

### Serum indexes and biomarkers

Currently, there is no suitable tumor marker that can predict the malignant transformation of endometriosis. In recent years, it has been reported that the serum CA125 level of patients with malignant transformation of endometriosis is normal [28–30]. The reasons may be as follows: (1) After the fibrosis of the malignant AWE tissue, the lesion was relatively limited and had little influence on the expression of hormones and various factors in vivo. (2) Due to the limitation of lesions, CA125 produced by ectopic endometrium is difficult to enter the blood circulation and has little influence on serum CA125. (3) The serum CA125 has low sensitivity to diagnose abdominal wall endometriosis. (4) Clinical reports show fewer cases overall, and there may be some accidents.

New data suggest that abnormal micrornas (miRNA) expression may play a part in the development of endometriosis [31]. In addition, a comprehensive analysis of miRNA expression in ovarian cancer and associated endometriosis revealed significant differences in miRNA expression [32]. These authors suggest that these miRNAs may be used as diagnostic or prognostic tools, but there is currently insufficient information about their targets, so their application in the diagnosis or treatment of endometriosis is not feasible at present. Malignant changes in endometriosis are usually hypothesized to be due to genetic mutations coupled with a relatively loose microenvironment [33]. EAOCs associated genes and signaling pathways include PTEN, CTNNB1, PIK3CA, SRC, KRAS, microsatellite instability and ARID1A [34, 35], which are known to play a crucial role in epithelial ovarian cancer relapse [36]. Loss of ARID1A expression is commonly associated with activation of the PI3K Akt pathway or amplification of XNF217, indicating an early event in the malignant transformation of endometriotic tissue to clear cell carcinoma [37]. A recent epigenetic study focused on a possible relationship between the methylation status of the promoter of the RASSF2A gene [38]. The study included 40 women who were underexpressed in both ovarian clear cell carcinoma(OCCC) and ovaria-endometrial cancer groups, and methylation status was bound up with clinical grade and stage, meaning that it may bloom into a reliable indicator of early detection. PTEN, a tumor suppressor gene significantly associated with ovarian cancer, is required to properly regulate the PI3K enzyme pathway [39]. This pathway is essential for the progression of endometriosis to ovarian cancer [40].

### Pathogenesis of endometriosis transforming into cancer

The mechanism of endometriosis malignant transformation depends on multiple factors such as heredity, immunity and environment. Complex interactions among hormonal influences, immune dysfunction and inflammation may promote malignant transformation of endometriosis. Xu et al. studied 12 cases of ovarian cancer arising from endometriosis and discovered some initial genetic abnormalities on chromosomes, such as sporadic loss of heterozygosity (LOH) may enhance the susceptibility to endometriosis. Xu et al. looked at 12 cases of ovarian cancer arising in endometriosis and found that the initial genetic abnormalities on some chromosomes such as sporadic loss of heterozygosity (LOH) may increase the predisposition to endometriosis [41]. Loss of heterozygosity and further genetic changes that accumulate at more sites may lead to aggressive features leading to the malignant transformation of endometriosis into cancer. These studies suggest that the malignant transformation of endometriosis can related to molecular genetics. It was reported that 13 (62%) of 21 patients with abdominal endometriosis malignant transformation had received estrogen replacement therapy [42]. According to a recent study, pathological examination after hysterectomy showed atypical hyperplasia of endometrium, which is caused by high estrogen or no antiestrogen and may be related to malignant transformation of abdominal endometriosis [43]. Almost all types of endometriosis patients have a lot of evidence of dysfunction of immune cells: T cell reactivity and NK cell toxicity declined [44]; Polyclonal activation production of B cells and antibodies has increased [45]; peritoneal macrophages increase in number and activate [46]; the apoptosis pathway was raised and inflammatory mediators were altered [47–50]. Free hemoglobin, heme and iron that are released in large quantities into the spaces between endometriosis cyst fluid during menstruation tend to oxidize and may spontaneously convert oxyhemoglobin to metHb. ROS (O2^−^) is constantly produced during the oxidation of hemoglobin. Iron derivatives can also provoke Fenton reaction and promote ROS (OH) production in endometriosis cysts. In addition, hemoglobin and heme activate the expression of various antioxidant genes. Heme by combining Bach1 directly or inducing NRF2 gene stimulate the antioxidant HO1 gene expression. Antioxidants are considered double-edged swords. Excessive ROS leads to cell death. Antioxidants by eliminating active oxygen ROS (O2and ˙OH) to reduce cell death, increasing cell survival and carcinogenesis [51–53]. In addition, endometrial lesions may produce more reactive oxygen species, and excessive toxic reactive oxygen species in the body can lead to the damage of proteins, lipids and DNA related to oxidative stress through oncogene mutation or damage [54]. Therefore, oxidative stress may be a key factor that causes DNA damage leading to the malignant transformation of endometriosis. Due to the limited case data of malignant changes in endometriosis, the exact etiology remains to be further research.

### Therapy

There is no standardized treatment regimens for this type of cancer, and the prognosis is difficult given the rarity of this type of malignancy. The consensus from case reports and reviews for both incisional endometriosis and malignant endometriosis is that complete resection of the tumor, uterus, and bilateral inguinal lymph nodes, with as wide and complete resection of the edge of the tumor and normal tissue as possible, and negative margins is optima1. After the operation, neoadjuvant chemotherapy and/or adjuvant radiotherapy were combined to guard against tumor relapse.

In this case, the lesion of abdominal wall endometriosis was first excised, and the pathological diagnosed low-differentiated endometrioid carcinoma of the abdominal wall after the operation. Postoperative follow-up was performed using 18-FDG PET-CT to evaluate the therapeutic effect and to determine the prognosis. Miller put forward some good prognosis factors: age under the age of 60, the tumor is small, good and without metastatic tumor differentiation, early removal of endometriosis lesions [55].

Our case is that of a postmenopausal woman, so there are no pain and periodic symptoms, only a gradual enlargement of the abdominal wall scar mass.Serum CA199 was increased and serum CA125 was normal, which was consistent with literature reports. Abdominal wall ultrasound was first performed and a cystic mass was found in the subcutaneous adipose layer of the right lower abdomen. Endometriosis was considered, but no malignant lesions were found. Due to the atypical clinical symptoms, it is very difficult to diagnose malignancy only by ultrasound examination, so we performed abdominal wall mass resection for the patient, and the postoperative pathologic diagnosis was low-differentiated endometrial carcinoma of abdominal wall.Therefore, in addition to routine abdominal ultrasound, MRI examination should also be used to determine the scope of lesions in patients with scar endometrioma.When abdominal endometriosis is not significantly improved after treatment and the mass rapidly increases, malignancy should be highly suspected and the disease stage should be determined by PET-CT examination.

In summary, malignant transformation of abdominal wall endometriosis is a infrequent condition associated with prior pelvic surgery, which should be attached great importance when scar lesions of abdominal endometriosis develop rapidly, and should be regarded as one of the differential diagnosis of abdominal mass after laparoscopic surgery. The diagnosis and stage of disease should be confirmed by clinical feature and imaging examination, and surgical resection should be carried out in combination with oncology principles and histopathological examination, combined with neoadjuvant chemotherapy and/or adjuvant radiotherapy. Although the current treatment is mainly radical surgery and adjuvant chemotherapy, the standardized treatment still needs further research. For most of the abdominal wall endometriosis associated with cesarean section, laparoscopic surgery. Therefore, It is a good way to prevent abdominal incision endometriosis is to reduce the cesarean section rate, protect the incision, take a specimen with an incision protection bag during the operation, reduce iatrogenic abdominal incision implantation and encourage breastfeeding to delay menstruation.

## Data Availability

The data supporting the conclusions of this article is available from corresponding author.

## Abbreviations

AFP: Alpha-fetoprotein
CA125: Carbohydrate antigen 125
HE4: Human epididymal protein 4
CEA: Carcinoembryonic antigen
CA199: Carbohydrate antigen 199
PAX-8: Not applicable
ER: Estrogen receptor
Ki-67: Not applicable
CD10: Not applicable
STATB2: Not applicable
GATA3: Not applicable
CDX-2: Not applicable
PR: Progesterone receptor
18-FDG PET-CT: 18-Fluorodeoxyglucose positron emission tomography
CT: Computed tomography
AWE: Abdominal wall endometriosis()
FGT: Female genital tract
IL: Interleukin
NK: Natural killer
M1: Not applicable
M2: Not applicable
SNP: Single nucleotide polymorphism
KB: Kilobyte
WNT4: The wingless-type mammalian mouse tumour virus integration site family member 4
GWAS: Genome wide association analysis
SNPs: Single nucleotide polymorphisms
ESR1: Not applicable
MRI: Magnetic resonance imaging
RNA: Ribonucleic acid
EAOCs: Not applicable
PTEN: Phosphatase and tensin homolog deleted on chromosome ten
CTNNB1: Catenin Beta 1
PIK3CA: Not applicable
Src: Sparse representation-based classifier
KRAS: Kirsten rat sarcoma viral oncogene
ARID1A: Not applicable
XNF217: Not applicable
RASSF2A: Ras association domain family 2A
OCCC: Ovarian clear cell carcinoma
XNF217: Not applicable
PIK3: Phosphoinositide 3-kinase
LOH: Loss of heterozygosity
ROS: Not applicable
NRF: Not applicable
DNA: Deoxyribonucleic acid
