# Correction to: Malignant transformation of endometriosis in a laparoscopic trocar site a case report

**DOI:** 10.1186/s12905-022-01896-7

**Published:** 2022-07-29

**Authors:** Ling Han, Bingyi Zhang

**Affiliations:** 1 Department of Obstetrics and Gynecology, The People’s Hospital of China Three Gorges University. The First People’s Hospital of Yichang, Jiefang Road 4, Yichang City, 443003 Hubei Province People’s Republic of China; 2 Department of Ultrasound Imaging, The People’s Hospital of China Three Gorges University. The First People’s Hospital of Yichang, Yichang City, Hubei Province People’s Republic of China

## Correction to: BMC Women’s Health (2022) 22:163 https://doi.org/10.1186/s12905-022-01749-3

Following publication of the original article [1], the affiliation of the author “Ling Han” was wrong and the correct affiliation is as follows ‘Department of Obstetrics and Gynecology, The People’s Hospital of China Three Gorges University. The First People’s Hospital of Yichang, Jiefang Road 4, Yichang City, 443003, Hubei Province, People’s Republic of China’.

The original article has been corrected.

## References

[1] Han L, Zhang B (2022). Malignant transformation of endometriosis in a laparoscopic trocar site a case report. BMC Women’s Health.

